# Could a fairer retirement age mitigate health inequalities? Evidence and decision-making

**DOI:** 10.3389/fpubh.2022.965140

**Published:** 2022-09-30

**Authors:** Chiara Ardito, Giuseppe Costa

**Affiliations:** ^1^Department of Economics and Statistics “Cognetti De Martiis,” University of Turin, Turin, Italy; ^2^Epidemiology Unit ASL TO3, Turin, Italy; ^3^LABORatorio R. Revelli – Centre for Employment Studies, Turin, Italy; ^4^NETSPAR – Network for Studies on Pensions, Aging and Retirement, Tilburg, Netherlands; ^5^Department of Clinical and Biological Sciences, University of Turin, Turin, Italy

**Keywords:** social determinants of health, inequality in health, life expectancies, retirement policy, inverse causality, direct causality, longevity inequality

## Increasing age of retirement

The interaction between health inequality and pension rules offers a good example of how scientific evidence may inform policy, highlighting the entry points of effective policy response to tackle and mitigate health inequality and its economic consequences.

The aging of the population has prompted governments around the world to take actions to ensure the sustainability of pension systems and public finances. National governments have responded to this demographic challenge by limiting access to retirement, raising the statutory pension age, and reducing economic incentives for early retirement. Tighter pension requirements and longer working life have led to a growing debate on the sustainability of work in old age, especially for those categories of workers exposed to more physically and psychologically demanding working conditions (1). Moreover, raising the statutory pension age in the same way for all workers ignores the existing differences in work capacity, health status, and life expectancy between professions that place disproportionally more to more vulnerable workers with potential detrimental consequences for their health (2).

In this paper, we review the reasons why we observe inequalities in health and longevity, the implications of their interaction with social security rules and pension outcomes, and finally we discuss some of the policy efforts needed to prevent and compensate health inequalities and their economic consequences.

## Growing evidence of unequal longevity

Inequalities in life expectancy are the most severe form of health inequality. In many European countries, differences in life expectancy at birth between people with a lower and a higher level of education, occupation, or income amount to between 5 and more than 10 years. Differences in healthy life expectancy often amount to even more than 15 years (3).

In the last few years, there has been an outspring of studies mapping how inequality in lifespan is evolving over time, adding a new temporal dimension to the phenomenon. Many recent prominent studies have highlighted that gains in life expectancy have not been equally distributed across socio-economic groups and that mortality gaps are widening in the USA [e.g., (4–6)]. The growing inequality in mortality at older ages has also been confirmed for several European countries by a recent special issue published by “Fiscal Studies” (7) on the evolution of mortality inequality from 1990 to 2019 in 12 Organization for Economic Co-operation and Development (OECD) countries. Replicating the method proposed by Currie and Schwandt (8) for the USA, the special issue suggests that, while in the youngest age group (0–4) inequality has decreased almost everywhere, among the elderly, for men and/or women, in most of the countries inequality is actually rising (USA, the UK, Spain, Portugal, Czech Republic, and The Netherlands) or remains stable (Canada and Finland).^1^ Paradoxically, the increasing inequalities in longevity among the elderly could be partly explained by a declined importance of the frailty bias (10) due to the success of prevention in reducing premature mortality also among the most vulnerable. Indeed, when mortality in midlife declines among individuals in low socioeconomic position, their chances to survive to older ages increase and social inequalities in mortality among the elderly could widen as result of changed compositions.

But what are the causes of such disparities? This pivotal question is still a matter of debate between different sciences, a situation that limits the possibilities for policymakers to take evidence-based decisions. Is the social that translates into biological (health inequalities represent the direct “causal” effect of socio-economic conditions), or is it the other way round, i.e., individuals with poorer health end up segregated at the bottom of the socio-economic gradient (inequalities represent the result of “selection” processes, or reverse “causation”)?

In an effort to assess the strength of the available evidence and adequacy of research methods, the European Federation of Academies of Sciences and Humanities (ALLEA) and the Federation of European Academies of Medicine (FEAM) launched a large interdisciplinary consultation of experts on health inequalities in the context of European countries and populations chaired by Johan Mackenbach. The final report represents the consensus reached and the related policy priorities (11). In substantive terms, the ALLEA and FEAM report concludes that the available evidence has led to some adequate insights into the direct causal effect of education on health, while for income and occupation, the picture of direct and reverse causation is more nuanced. The available counterfactual evidence does not seem to show that income directly affects physical health in adulthood in high-income countries, while there is evidence of a causal effect of parental income on children's health. At the same time, there is also evidence that reverse causality may play a limited role in educational, income, and occupational inequalities in health. Moreover, unequal exposure or susceptibility to different risk factors (such as smoking, work, and housing conditions) or unequal social vulnerability to the experience of diseases are among the mechanisms that mediate or moderate the unequal impact of health on education, income, and occupation for which the evidence is well assessed.

The most challenging aspect for research is the fact that these mechanisms interact with each other in recursive relationships and feedbacks throughout the life course (and even intergenerationally) with multiple causal pathways and long causal chains, eventually making it difficult to assess the relative importance of direct and reverse causation and of each potential generating mechanism.

## Interaction of longevity inequality and retirement policies

Despite the huge advancements made in prevention in recent decades, work trajectories end up around the statutory retirement age with unequal work ability and unequal residual life expectancy, due to unequal exposure to well-known and still unrecognized occupational risk factors, unfavorable living conditions, and unhealthy behaviors that are often adopted as an adaptative response to chronic distress. This is the legacy that health inequalities leaves for retirement policies, which should decide, regardless of the direction of the causal mechanism, if and how to compensate for this final health disadvantage that gives rise to a greater economic disadvantage.

Indeed, longevity inequalities interfere with pension policies which induces a redistribution of resources from the less well-off to the better-off. The mechanism is intuitive and has been recently highlighted by several OECD reports and academic works. First, life expectancy at retirement *on average* translates into the number of years of pension receipt. Second, life expectancy is a key parameter in pension rules and determines eligibility and benefits amounts in several countries. Approximately two-thirds of OECD pension systems employ automatic adjustment mechanisms, including, for example, linking benefits to life expectancy at retirement through longevity factors in notional defined contribution schemes^2^ and linking the statutory retirement age to life expectancy changes registered in the population (12).^3^

Thus, individuals with lower-than-average life expectancy receive the pension benefit for a shorter period of time, experiencing a loss of pension wealth relative to what is actuarially fair. In contrast, groups with above-average life expectancy will benefit from a pension “premium,” financed by the most disadvantaged groups (12–14). Bommier et al. (15) estimated that occupational inequality in mortality neutralizes approximately one-third of the income redistribution built into the French PAYG pension system, while Whitehouse and Zaidi (16) suggest that it fully offsets the redistribution in Germany similar to what is estimated for the USA (17). OECD (13) points out that the 3-year average gap in remaining life expectancy at retirement reduces total pensions received by low earners by 13% relative to those of high earners, on average across countries, beyond the effects of lower earnings.

## Policy implications

Should policymakers take health inequality and their consequences into account, even though scientific evidence is not yet able to completely disentangle the causal pathways linking low socioeconomic position to shorter life expectancy?

We suggest that the existing evidence on large and increasing longevity gaps, and their potential impact on pension wealth inequality, justifies efforts to implement specific measures to counteract and prevent pension systems from leading to regressive redistribution.

This calls for the triangulation of interventions aimed at addressing both the direct causality mechanisms that generate health inequalities—with implications for interventions aimed at preventing unfavorable working and material conditions from leading to unequal life expectancy—and reverse causality ones—involving compensatory interventions for unequal pension treatments depending on differential life expectancy.

On one hand, preventive interventions aimed at limiting the insurgency or progression of health inequality involve both workplace intervention aimed at improving organizational, physical, and psychosocial working conditions, the promotion of healthier lifestyles in the workplace, to slow down the decline in work capacity, and structural interventions that improve access to and quality of health care, education, welfare assistance to tackle the long chain that—if not entirely causing—at least sustains and maintains health inequalities. To promote longer and healthy working lives, several measures have been widely suggested. These include workplaces accommodation by improving working conditions, the accommodation of tasks and the working environment across the working life depending on the workers' actual work capacity, increasing flexible time schedules, with reduction, or elimination of shift work and transfer to more light work or tutoring tasks (18, 19). However, companies are, in general, reluctant to job tailoring approaches and modifications of work organization in front of an aging workforce as these measures require significant investments. A substantial proportion of companies do not have enough resources to adjust their workplaces, and are more likely to facilitate early retirement openings for older workers, somehow externalizing the costs of managing an aging workforce.

This brings us to the other side of the story, i.e., to the policies that ought to compensate for the presence of health inequalities and to buffer their potentially adverse economic consequences (i.e., the reverse causality side). Based on recent findings on the possible harmful effect of retiring at an older age for vulnerable workers and on the loss of pension wealth induced by longevity inequality, the obvious policy response is to increase the flexibility around the age at which individuals can retire, ensuring that lower socioeconomic groups are not penalized in retirement due to having poorer health and shorter life expectancies. This can be implemented by offering differentiated retirement ages and by strengthening disability or long-term unemployment benefit schemes for older workers whose career is cut by disability or reduced employability. However, in pension systems that adopt the actuarial correction for retirement age, the option of early retirement should be accompanied by a reform of the pension formula to take into account the difference in life expectancy between socioeconomic groups, by adopting group-specific longevity factors used to calculate pension annuities to ensure actuarial fairness also for low socioeconomic group individuals with shorter life expectancy (20).

While all OECD countries offer access to disability and unemployment benefits for those whose career is impeded by ill health (14), the coverage and generosity vary widely across countries, with concerns about their capacity to provide sufficient financial support for healthy living to workers who, because of their reduced work ability, are expelled from the labor market (21). To our knowledge, there are no experiences around the world of systematic linking retirement ages or longevity factors to occupation-based (or education- or income-based) life expectancies. Nevertheless, the question is now entering scientific and public debate (13, 14, 22). The closest experience is that of special retirement rules for arduous and hazardous jobs. Approximately two-thirds of European countries allow early retirement without benefit penalty for selected and specific groups of workers, such as those in arduous and hazardous jobs (23). The definition of which jobs to include in this category is a political challenge. For instance, it is difficult to account for mixed careers with periods of arduous and non-arduous work and it is not easy to define arduous work unambiguously, which requires the convergence of experts and social partners' opinion. In their overview, Natali et al. (23) find a wide variety of classifications based on lists of conditions/occupations/sectors that vary across countries, noting that the concept is often extremely rigid, so that only a small minority of workers is covered.

Another compensatory mechanism could be the adoption of a pension scheme for survivors. Survivor pensions, by ensuring that the surviving partner will continue to receive a portion of the deceased's pension, could help mitigate the disadvantage faced by individuals from low socio-economic groups who tend to spend a shorter time in retirement relative to their working time and receive a lower “return” for their contributions paid into the pension system. However, despite the fact that most OECD countries still have mandatory survivor pensions, in recent decades the trend has been to tighten or eliminate these schemes (14) and of all NDC countries, only Italy and Greece have a survivor's pension (12). However, the survivor pension scheme also has limitations: it provides unequal treatment to persons depending on whether or not they live in a couple, and it runs the risk to consolidate a regressive redistribution of resources given that survivors tend to show inequalities in longevity similar to their partners due to educational and assortative income mating (24).

Finally, an additional compensatory instrument for economic disadvantages induced by health impairments, is progressivity. Increased progressivity of the public pension system and tax rules with lower marginal tax rates for low incomes, can significantly help mitigate or eliminate relative inequalities in pension outcomes across socioeconomic groups (14, 20).

## Conclusions

A very simple fact, unequal longevity, has several implications in different policy domains, such as the way economic activities and industries shape working conditions and the way pension regimes distribute their benefits. The remaining uncertainties about the role of low socioeconomic position in explaining unequal longevity through direct and reverse causation mechanisms do not exempt preventive and compensatory policies to search for more equal opportunity for health in each step of the working and retirement trajectory of a person. This is a good example of how policies should learn to wear the health equity lens whenever and wherever possible to make fairer choices, as recommended by the Health Equity in All Policies approach.

## Author contributions

All authors listed have made a substantial, direct, and intellectual contribution to the work and approved it for publication.

## Funding

This work was supported by the Italian Ministry of Health funding Project No. RF-201602364270.

## Conflict of interest

The authors declare that the research was conducted in the absence of any commercial or financial relationships that could be construed as a potential conflict of interest.

## Publisher's note

All claims expressed in this article are solely those of the authors and do not necessarily represent those of their affiliated organizations, or those of the publisher, the editors and the reviewers. Any product that may be evaluated in this article, or claim that may be made by its manufacturer, is not guaranteed or endorsed by the publisher.

## Footnotes

1 The authors of this paper recently studied the evolution of life expectancy and mortality differentials among private sector employees in Italy. Their analyses confirm that longevity inequality among the elderly is widening in Italy, too (9).

2 In a notional defined contribution pension system, the amount of pension benefit is defined establishing the equality between individual's contributions (plus interests) and the individual's future expected benefits through conversion factors that depend on life expectancy at retirement, i.e. the so-called longevity factors.

3 The link can be a one-to-one, meaning that a 1-year increase in life expectancy translates into a 1-year increase in the statutory retirement age (e.g., Italy, Denmark, Estonia, and Greece), or a two-thirds link (e.g. Finland, The Netherlands, and Portugal).
